# Sensitivity of contact-tracing for COVID-19 in Thailand: a capture-recapture application

**DOI:** 10.1186/s12879-022-07046-6

**Published:** 2022-01-29

**Authors:** R. Lerdsuwansri, P. Sangnawakij, D. Böhning, C. Sansilapin, W. Chaifoo, Jonathan A. Polonsky, Victor J. Del Rio Vilas

**Affiliations:** 1 Department of Mathematics and Statistics, Faculty of Science and Technology, Thammasat University, Pathum Thani, Thailand; 2 Southampton Statistical Sciences Research Institute and Mathematical Sciences, University of Southampton, Southampton, UK; 3 Department of Disease Control, Ministry of Public Health, Nonthaburi, Thailand; 4 World Health Organization, Geneva, Switzerland; 5 World Health Organization, World Health Emergencies, South East Asia Regional Office, New Delhi, India

**Keywords:** COVID-19, Contact tracing, Thailand, Capture-recapture, Sensitivity

## Abstract

**Background:**

We investigate the completeness of contact tracing for COVID-19 during the first wave of the COVID-19 pandemic in Thailand, from early January 2020 to 30 June 2020.

**Methods:**

Uni-list capture-recapture models were applied to the frequency distributions of index cases to inform two questions: (1) the unobserved number of index cases with contacts, and (2) the unobserved number of index cases with secondary cases among their contacts.

**Results:**

Generalized linear models (using Poisson and logistic families) did not return any significant predictor (age, sex, nationality, number of contacts per case) on the risk of transmission and hence capture-recapture models did not adjust for observed heterogeneity. Best fitting models, a zero truncated negative binomial for question 1 and zero-truncated Poisson for question 2, returned sensitivity estimates for contact tracing performance of 77.6% (95% CI = 73.75–81.54%) and 67.6% (95% CI = 53.84–81.38%), respectively. A zero-inflated negative binomial model on the distribution of index cases with secondary cases allowed the estimation of the effective reproduction number at 0.14 (95% CI = 0.09–0.22), and the overdispersion parameter at 0.1.

**Conclusion:**

Completeness of COVID-19 contact tracing in Thailand during the first wave appeared moderate, with around 67% of infectious transmission chains detected. Overdispersion was present suggesting that most of the index cases did not result in infectious transmission chains and the majority of transmission events stemmed from a small proportion of index cases.

## Background

Following the notification of the first COVID-19 cases in Thailand on 11 January 2020, the Department of Disease Control (DDC), Ministry of Public Health Thailand started recording essential information to monitor the epidemic. By early May 2020, the epidemic had receded from a daily peak of 188 cases in mid-March 2020 to single digit daily counts. The first wave of the epidemic was under control. At the time of writing Thailand was experiencing a second wave that started in early December 2020, with a cumulative number of just over 26,000 cases as of 5 March 2021 (https://ddc.moph.go.th/viralpneumonia/eng/index.php).

Thailand’s successful initial response to COVID-19 was aided by a strong national capacity to trace and quarantine contacts using Rapid Response Teams and Village Health Volunteers who were trained during earlier major infectious disease outbreaks such as H1N1, SARS, and Avian Influenza [1, 2]. Despite the prompt reaction by local health authorities, the Intra-Action-Review (IAR) on Thailand’s response to COVID-19 highlighted the need for a sensitive COVID-19 surveillance system to facilitate detection of individual cases, small clusters and monitor trends [3].

Contact tracing (CT) aims to identify, assess and manage contacts exposed to disease to prevent onward transmission [4]. In this capacity, CT remains a critical function towards the control of infectious diseases. Similar to other surveillance efforts, sensitivity, or the ability to detect all the events of interest, is one of the most relevant technical attributes towards the assessment of CT performance [5]. For COVID-19, timeliness and sensitivity are the most cited performance attributes [6]. Whereas timeliness can be directly measured (and it is normally decomposed in multiple metrics to reflect the many steps in the flow of information and biological samples that constitute the surveillance system), that is not the case for sensitivity. Several approaches towards its estimation have been suggested [5, 7]. Here we focus on capture-recapture (CRC) models [8, 9]. Broadly, this family of methodological approaches estimates the number of individuals missing from identifying mechanisms such as disease surveillance systems (SS). The estimation of the SS sensitivity and probability of event detection follows.

CRC approaches have been extensively used to estimate disease SS sensitivity [10]. Specifically on CT, Polonsky and colleagues applied uni-list CRC models to Ebola Virus Disease (EVD) data from the 2018–2020 EVD outbreak in North Kivu Province, Democratic Republic of the Congo (DRC) [11]. The authors addressed two specific questions: (1) what is the true number of index cases with unobserved contacts (in effect assessing the sensitivity of contact identification efforts), and (2) what is the true number of index cases with secondary cases among their contacts (in effect assessing the sensitivity of case detection among contacts). CRC approaches, on country aggregated case data, were also applied to estimate the true number of COVID-19 infections, estimated to be three to eight times larger than those reported [12].

Here we first describe Thailand’s first wave of COVID-19 CT data, and then the application of uni-list CRC models to quantify the number of unobserved index cases, and CT sensitivity. Specifically, we aim to answer the following: question (1) how many index cases with contacts were missed by CT, and question (2) how many index cases with infected contacts were missed by the CT mechanism.

## Methods

### Materials

**Fig. 1:**
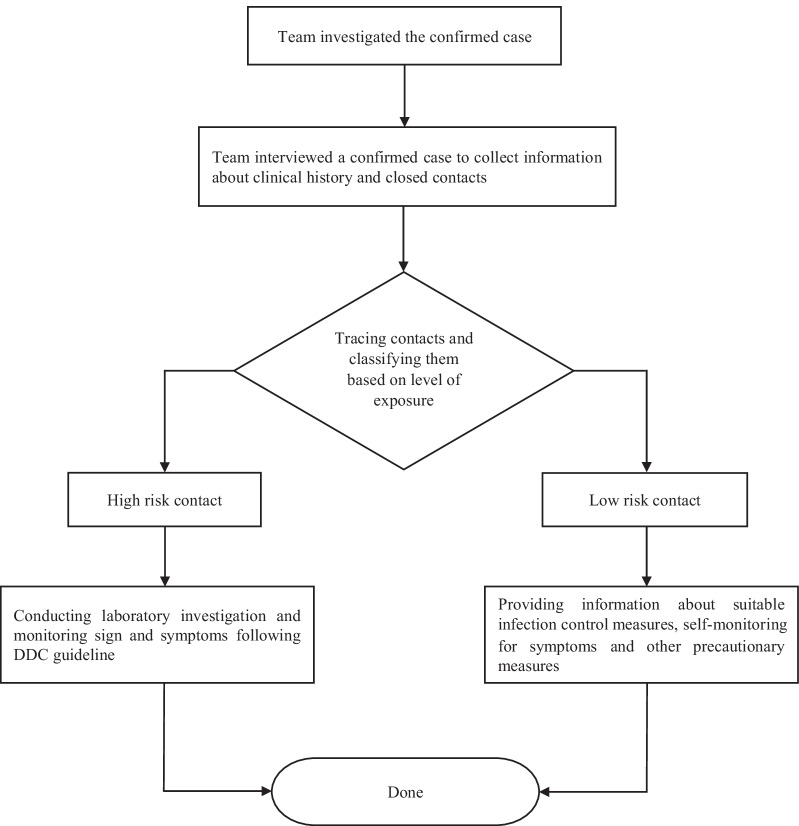
A brief flowchart of the contact tracing process undertaken by the local communicable disease control units (CDCU) and the Department of Disease Control (Compiled by the authors)

Our data stems from Thailand’s regular COVID-19 CT operations. Figure 1 presents a flowchart of the contact tracing process undertaken by the local communicable disease control units (CDCU) and joint investigation teams (JIT) from DDC. Once the patient is diagnosed as being infected with SARS-CoV-2, so called the confirmed case, contact tracing will be conducted to obtain the list of contacts. The identified contacts are classified as either high-risk contacts or low-risk contact following investigation guidelines [13]. High-risk contact is defined as a contact who is more likely to contract the virus through exposure to respiratory secretions of the confirmed case while not wearing PPE according to standard precautions. Low-risk contact is defined as a contact who is less likely to contract the virus from the confirmed case. This includes contacts who have not met the definition for high-risk contact. Only high-risk contacts were quarantined in the designated places and basic demographic information such as age, sex, and nationality were collected and recorded in the contact form. Our data set comprises the period 11 January 2020 to 30 June 2020. A total of 352 cases were identified through contact tracing system leading to 6359 high risk contacts and 4299 low risk contacts.

### Initial analysis

We describe the data according to the available demographic predictors associated with the index cases (age, sex, and nationality) and the number of contacts per index case. We applied logistic regression to assess whether any of the above predictors (with age as a continuous variable), had any effect on the probability of identifying secondary cases. Using the best fitting models of the count distributions (see next section), we regressed the observed covariates on the number of contacts per index case with at least one contact (*n* = 341) to assess whether we should adjust for covariates in our capture-recapture calculations. We also applied a zero-inflated negative binomial model. By the full distribution of all index cases we mean the following: out of the 352 index cases only 30 had infectious contacts (secondary infections), namely 16 index cases had 1 infected contact, 9 had 2, 4 had 3 and 1 had 4 infectious contacts. The large number of zeros is reflected in the zero-inflated negative-binomial modeling which adds simply an additional parameter just for those with zero infectious contacts.

### Capture-recapture modelling

We are interested in deriving an estimate of the unknown true number of COVID-19 cases with contacts that entered the CT mechanism. This would address question 1 (Q1) as above. The tracing of contacts is likely to lead to the identification of secondary infections for a subset of index cases. This data informs question 2 (Q2). For both questions, the data can be binned into the number of index cases with one listed (Q1) or infected (Q2) contact ($f_1$), two listed or infected contacts ($f_2$), and so on up to the number of index cases with the maximum number of listed or infected contacts ($f_m$). Here, $f_0$, the frequency of index cases with unobserved contacts (for Q1) or unobserved infected contacts (for Q2) is unknown and the target of the inference. Statistically, the identification process leads to a zero-truncated count distribution of cases with at least one listed or infected contact, i.e. with positive integers (ones, twos, threes, etc.), but no zeros. By applying CRC approaches, we can infer $f_0$, the number of unobserved cases with at least one listed or infected contact.

For both questions, we fit parametric models (Poisson, Negative Binomial, and Geometric) to the observed counts using the maximum likelihood method. Then, the smallest Akaike and Bayesian Information Criterion (AIC and BIC, respectively) are used for model selection. After estimating model parameters, we can estimate $f_0$ as

1 $$\begin{aligned} {\hat{f}}_0 = \frac{np_0}{1-p_0}, \end{aligned}$$

where *n* is the observed sample size and $p_0$ is the estimated probability of missing an index case with non-zero contacts as computed from the models. The population size estimator ${\hat{N}}=n+{\hat{f}}_0$ follows.

In addition to the model-based estimators we consider two further alternatives for comparison purposes: the Turing’s estimator [14] and Chao’s lower bound estimator [15]. Turing’s estimator is formulated under a homogeneous Poisson distribution with parameter $\lambda$. Let $p_0$ be the probability of zero count or missing an observation. We have

2 $$\begin{aligned} p_0 = e^{-\lambda } = \frac{e^{-\lambda }\lambda }{\lambda } = \frac{p_1}{\lambda }. \end{aligned}$$

The estimate of $p_0$ can be calculated from observed frequencies as follows

3 $$\begin{aligned} {\hat{p}}_0 = \frac{f_1/N}{S/N} = \frac{f_1}{S}, \end{aligned}$$

where $S = \sum _{i=0}^m if_i$. Thus, Turing’s estimator for estimating the population size is given by

4 $$\begin{aligned} {\hat{N}}_{Turing} = \frac{n}{1-f_1/S}. \end{aligned}$$

Chao (1987) suggested a mixed Poisson model with $p_i = \int _0^{\inf } \frac{e^{-\lambda }\lambda ^i}{i!}g(\lambda )d\lambda$ for *i* = 0, 1, 2, ... and arbitrary density $g(\lambda )$ [15]. Chao’s estimator incorporates not only the unobserved heterogeneity in the Poisson parameter but also leads to a very simple nonparametric estimator by applying the Cauchy–Schwarz inequality to the lower bound for the probability of a not observed event

5 $$\begin{aligned} p_0 \ge \frac{p_1^2}{2p_2}. \end{aligned}$$

Replacing these probabilities by observed frequencies, the lower bound for the estimate of zero counts is computed as ${\hat{f}}_0 \ge f_1^2/(2f_2)$. As a result, Chao’s lower bound estimator for the population size is

6 $$\begin{aligned} {\hat{N}}_{Chao} = n + \frac{f_1^2}{2f_2}. \end{aligned}$$

Clearly, (6) uses only part of the available information, $f_1$ and $f_2$, as opposed to Turing estimator that uses all the information in the sample by means of *S*. In addition, a mixing distribution $g(\lambda )$ is not required to be specified and estimated showing the non-parametric nature of this estimator.

### Confidence Interval for the unknown population size

To estimate 95% confidence intervals (95% CIs), we use resampling techniques as described in the CRC literature [16, 17]. Suppose that ${\hat{N}}$ is the estimated size under a fitted model. Then, we generate *B* samples of size ${\hat{N}}$ using the fitted model and its estimated parameter(s). For each sample, all zeros are truncated and the size estimate ${\hat{N}}_b$ computed, for each of the samples *b* = 1, 2,..., *B* leading to a sample of estimates ${\hat{N}}_1$, ${\hat{N}}_2$,..., ${\hat{N}}_B$. We choose *B* = 10,000 to minimize bootstrap simulation random error, and then use two methods towards CI construction:

- The normal approximation method, using the median a robust estimator for the mean where $\bar{{\hat{N}}}$ = median(${\hat{N}}_b$| *b* = 1, 2,..., *B*) and calculate the bootstrap standard error as
  7 $$\begin{aligned} SE = \sqrt{{\text {median}}(({\hat{N}}_b - \bar{{\hat{N}}})^2| b = 1, 2,\ldots , B}). \end{aligned}$$
  The 95% confidence interval for the true population size can then be constructed by means of ${\hat{N}} \pm 1.96 \times SE$.
- The percentile method where we use the 2.5th percentile of the distribution of ${\hat{N}}_b$ as the lower end and the 97.5th percentile as the upper end.

## Results

### Descriptive analyses

**Fig. 2:**
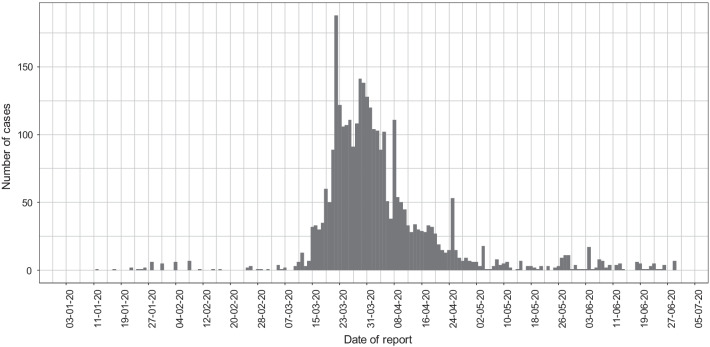
Epidemic curve of the COVID-19 outbreak in Thailand from 4 Jan 2020 to 30 June 2020 (3171 confirmed cases)

In the period 4 Jan 2020 to 30 June 2020, 3171 cases were confirmed (Fig. 2). Of those, 352 (11.1%) index cases were followed through CT leading to the identification of subsequent contacts for 341 of them. Among these 341 index cases with non-zero contacts from which 6,359 high risk contacts were listed, there were 44 index cases with one contact ($f_1$ = 44), 22 with two contacts ($f_2$ = 22), 24 with three contacts ($f_3$ = 24), and so on. Table 1 shows the complete distribution of index cases with traced contacts for the first 50 index cases. For infected contacts, the complete distribution is as follows: index cases with one infected contact ($f_1$ = 16), two infected contacts ($f_2$ = 9), three infected contacts ($f_3$ = 4), and four infected contacts ($f_4$ = 1).

**Table 1:** Frequency distribution of counts of index cases with contacts (only first 50 counts)

$f_1$	$f_2$	$f_3$	$f_4$	$f_5$	$f_6$	$f_7$	$f_8$	$f_9$	$f_10$
44	22	24	16	15	10	11	10	9	9
$f_{11}$	$f_{12}$	$f_{13}$	$f_{14}$	$f_{15}$	$f_{16}$	$f_{17}$	$f_{18}$	$f_{19}$	$f_{20}$
9	10	6	7	8	16	3	2	3	8
$f_{21}$	$f_{22}$	$f_{23}$	$f_{24}$	$f_{25}$	$f_{26}$	$f_{27}$	$f_{28}$	$f_{29}$	$f_{30}$
5	3	5	6	1	6	1	2	4	5
$f_{31}$	$f_{32}$	$f_{33}$	$f_{34}$	$f_{35}$	$f_{36}$	$f_{37}$	$f_{38}$	$f_{39}$	$f_{40}$
4	4	1	3	2	2	1	1	1	6
$f_{41}$	$f_{42}$	$f_{43}$	$f_{44}$	$f_{45}$	$f_{46}$	$f_{47}$	$f_{48}$	$f_{49}$	$f_{50}$
0	3	1	1	1	2	0	1	0	2

Of the 341 index cases with at least one contact, 196 (57.48%) were males and 145 (42.52%) were females. At a 5% level of significance, there was sufficient evidence to conclude that there was a difference between the proportions of these contacts from male and female (goodness-of-fit Chi-square test with P-value = 0.007). See more details of the goodness-of-fit test in [18]. The median age was 37 years (mean = 39.62, interquartile range (IQR) = 28–50, min = 0.3, max = 83). The statistics of age by gender are given in the following:

**Table:** 

	Age of male	Age of female
Median	40	34
IQR	29–52	26–47

These showed median age for males was significantly greater than that of females (Wilcoxon signed-rank test with P-value = 0.004) [18]. The vast majority of cases (290, 85.04%) cases were Thai. Meanwhile, 51 (14.96%) were foreign nationals: 26 cases (7.62%) from China, 5 cases (1.46%) from Japan, 4 cases (1.17%) from Denmark, and 61 cases from other locations.

From 341 index cases with non-zero contacts noted before, 30 (8.8%) index cases had at least one infected contact. The median age of this set of index cases was 44 years (mean $= 42.87$, IQR $= 29.25$–56, min $= 6$ and max $= 80$). Summary statistics of age by gender are concluded as follows:

**Table:** 

	Age of male	Age of female
Median	45.5	36
IQR	38.5–46.75	28.25–46.75

Furthermore, almost all index cases with infected contacts were Thai (28 cases, 93.33%). We also show summary statistics for the set of 30 index cases with infected contacts in Table 2.

**Table 2:** Summary statistics for the 30 index cases with infected contacts

Number of non-zero contacts	1	2	3	4
Frequency	16	9	4	1
Median age	40.5	41	59.5	66
Minimum age	6	20	26	66
Maximum age	64	80	68	66
95% CIs for mean of age	32.4–48.42	26.24–53.76	23.47–83.03	66
%Female	50%	55.56%	25%	0

A zero-truncated Poisson regression (best fitting model for this reduced dataset (30 observations)) showed no significant covariate effects on the number of contacts per index case with at least one secondary case. For the larger dataset of the number of contacts per index case, the best fitting model (a zero-truncated negative binomial regression) also showed no significant effects of the covariates. These results support that no consideration of observed heterogeneity in our capture-recapture models was required. The logistic regression showed no significant covariate effects either.

The application of a zero-inflated negative binomial model to the full distribution of all index cases (*n* = 352 that includes all cases detected through the CT mechanism and their contacts; we note that for 311 of such cases there were zero infected contacts, hence the use of a zero-inflated model) allows the estimation of the average number of secondary infections over the course of the outbreak that equates to an average effective reproductive number (RE = 0.14; 95% CI: 0.09–0.22), and dispersion parameter (*k* = 0.1). We note that our estimate of the reproduction number applies to the entirety of the period under study and is sensitive to the implementation of several public and health social measures in country at different times.

### Applications of capture-recapture models towards the estimation of contact tracing sensitivity

**Fig. 3:**
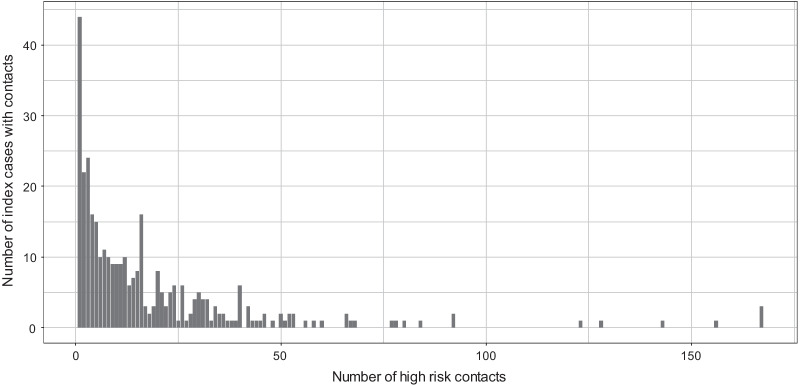
Frequency distribution of number of index cases with contacts (*n* = 341)

**Fig. 4:**
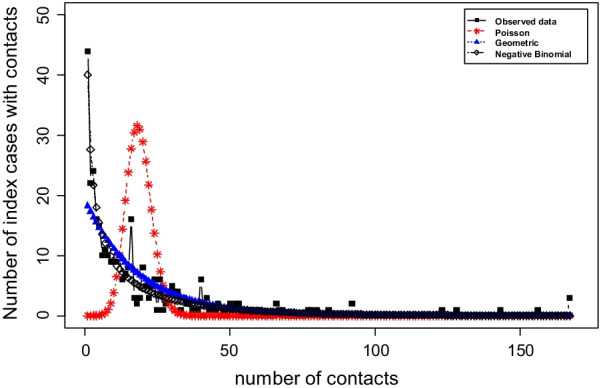
Frequency distribution of number of index cases with contacts (*n* = 341)

As seen in Fig. 3, the distribution of index cases with contacts presents a long tail. Clearly, this long-tailed distribution is fitted a lot better by the negative binomial than by the Poisson distribution and the geometric distribution (see Fig. 4). The best fit (in effect addressing Q1) is given by a zero-truncated negative binomial model (Table 3) leading to an estimate of unobserved index cases with contacts of ${\hat{f}}_0$ = 98.18, and an estimated size of the overall count of index cases with contacts of ${\hat{N}}$ = 439.18. In the appendix we derive population size estimators for Turing and Chao approaches for the chosen negative binomial distribution, and in Table 4 we present the results of the three estimators including 95% confidence intervals. Note that Chao’s estimator is slightly higher than the other two, indicating potential residual heterogeneity. However, this might be also still within random error variation as the confidence intervals in Table 4 express. Using the estimated $f_0$ from the zero-truncated negative binomial model, we estimate the CT sensitivity to detect index cases with contacts as 341/(341 + 98) = 0.776 or 77.6%.

**Table 3:** Model results and fit criteria for the count data of index cases with all contacts

Model	Log-likelihood	AIC	BIC
Poisson (${\hat{\lambda }}$ = 18.648)	$-$4663.285	9328.569	9332.401
Negative Binomial (mue = 14.479, size = 0.4197)	$-$**1304**.**133**	**2612**.**266**	**2619**.**93**
Geometric (${\hat{p}}$ = 0.0536)	$-$1329.368	2660.735	2664.567

Bold values indicate that Negative Binomial is the best fit for the distribution of the number of index cases with contacts (n=341) due to the smallest AIC and BIC

**Table 4:** Estimates of unobserved contacts, population size and 95% CI (*n* = 341) for the three approaches (based upon the negative-binomial model)

Estimators	${\hat{f}}_0$	${\hat{N}}$	Bootstrap median	95% CI normal approximation	CI from percentile BT
MLE	98.18	439.18	438.271	402.07–474.47	391.25–510.05
Turing	101.78	442.78	438.0309	401.35–474.72	390.74–511.09
Chao	148.84	489.84	439.5344	382.01–497.06	376.29–566.81

Fifth and sixth column show bootstrap CI by the normal approximation and percentile methods, respectively

**Fig. 5:**
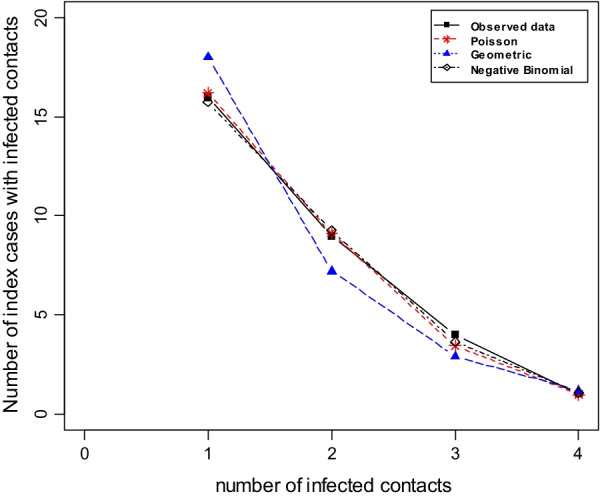
Frequency distribution of number of index cases with infected contacts (*n* = 30)

Next, we address Q2. As can be seen in Fig. 5, the best fitting model is given by the zero-truncated Poisson model (Table 5) with ${\hat{\lambda }}$ = 1.126. Table 6 provides the estimated frequency of index cases with infected but unobserved contacts for the zero truncated Poisson ${\hat{f}}_0 = \frac{n e^{-{\hat{\lambda }}}}{1-e^{-{\hat{\lambda }}}}$ = 14.37, and those from the Turing and Chao approaches for reference. Using the estimated $f_0$ from the zero truncated Poisson model we estimated the sensitivity of contact tracing to detect index cases with infected contacts as 30/(30 + 14) = 0.676 or 67.6%.

**Table 5:** Model performance for the count index cases with infected contacts

Model	Log-likelihood	AIC	BIC
Poisson (${\hat{\lambda }}$ = 1.126)	$-$**32**.**6684**	**67**.**3368**	**68**.**738**
Negative Binomial (mue = 1.176, size = 1175.709)	$-$32.6914	69.3829	72.1853
Geometric (${\hat{p}}$ = 0.6)	$-$33.6506	69.3012	70.7024

Bold values indicate show that Poisson is the best fit for the distribution of the number of index cases with infected contacts (n=30) due to the smallest AIC and BIC

**Table 6:** Estimates of unobserved index cases with infected contacts, population size and 95% CI (*n* = 30) for the three approaches (based upon the Poisson model)

Estimators	${\hat{f}}_0$	${\hat{N}}$	BT median	95% CI	CI from percentile BT
MLE	14.37	44.38	45.51	36.62–54.40	33.87–64.17
Turing	14.12	44.12	45.47	36.01–54.94	33.29–64.63
Chao	14.22	44.22	45.5	33.21 $-$57.79	31.64–77.40

Fifth and sixth column show bootstrap CI by the normal approximation and percentile methods, respectively

## Discussion

Our results show a moderately sensitive CT system in Thailand, able to detect more than two thirds of infectious transmission chains during this first wave. The capacity of the system to detect index cases with at least one contact was even higher at 77.6%. Further, it was straightforward to estimate the average intensity of the transmission; this appeared low as shown by the estimated RE (0.14; 95% CI: 0.09–0.22). As reported by an increasing number of works [19, 20], we have also found substantial overdispersion in our data suggesting that most of the index cases did not result in infectious transmission chains and the majority of transmission events stemmed from a small proportion of index cases.

The magnitude of the unobserved fraction of COVID-19 cases has been estimated as substantial. Here we propose a mechanism towards the estimation of such undetected population but stress that as the unit of study is the index case once they enter the CT mechanism, which allows the repeated identification of the index case through his/her contacts and the subsequent generation of the count distributions of interest, our inference is therefore limited to CT. In other words, we cannot estimate the overall size of under-reporting that may be associated with other forms of COVID-19 surveillance. Moreover, reiterating the index case as our unit of inference, our results cannot inform the number of contacts (infected or not) missed by CT, from the missed number of index cases estimated by our models. Other approaches have recently been suggested towards the estimation of the under-reported fraction of COVID-19 cases. Lawson and Kim (2021) have recently modelled the spatio-temporal distribution of COVID-19 in South Carolina (US) and considered the role of asymptomatic transmission as a latent effect, and suggested the use of scaling factors to account for the missing cases as done for seasonal influenza [21]. As our data did not specify whether the index cases were symptomatic or not, our estimates of $f_0$ are likely to include both.

### Statistical considerations

For each index case, the number of observed contacts allowed to derive a count distribution which has then been modelled parametrically. Using the best fitting model, the number of index cases with unobserved contacts could be determined and, thus, the completeness of CT. Clearly, the estimate of the frequency of index cases with undetected contacts depends on the model of choice. Hence, we also considered alternative estimators including those of Chao and Turing which weaken the assumption of the chosen model. Chao’s estimator allows for heterogeneity in the parameter of the probability model whereas Turing’s estimator avoids maximum likelihood estimation. If these alternative approaches lead to substantially different estimates of the size, the choice of the model might be questionable. In all our analyses, the approaches led to similar size estimates. We have also considered whether the distribution was affected by the observed heterogeneity as captured by the available covariates gender, age, or nationality. A generalized linear model analysis (using Poisson and logistic regression) showed no significant association to any of these covariates. Hence, we did not consider a stratified capture-recapture modeling. This is not to say that these variables have no effect on the sensitivity of CT, just that for our dataset such predictors did not show any significance in the unobserved number of index cases. We note that a recent study on EVD showed different patterns in the number of contacts and the probability of zero contacts between two well-defined waves in DRC, and suggested possible improvements in CT as teams become more accustomed over time [11]. In our case, there was no clear break in the time series of cases to support such analysis. However, comparing first and subsequent waves of cases in Thailand would be feasible.

We assumed a closed population which is a reasonable assumption under lock-down conditions, and typically met in these kinds of applications by steering the observational window to be small enough. We also assumed independence in the observation (sampling) of index cases. This would be typically violated if these would occur in clusters. Heterogeneity and clustering work in the same way so that Chao’s lower bound estimator would still be a conservative approach to the estimation of completeness. In all cases, the parametric modelling and Chao’ estimator have returned similar findings which supports our assumption of independence.

### Perspectives

Several countries have used different CT mechanisms, e.g., traditional CT, use of CCTV systems, mobile applications, for the purpose of identifying contacts. In such situations, multi-list CRC models might merit study to assess multiple identification of contacts by more than one data stream.

Hook and Regal (1995) stated that the application of CRC methods had very little impact in the public health arena. In other words, their policy value might be small [22]. Providing more informative outputs with indication of where under-reporting is occurring, and what population groups might be more affected would increase the policy value [23]. However, our limited dataset did not present significant heterogeneity to inform such questions. Richer datasets would be required to that effect. A related challenge is the timing of these types of evaluations, with their retrospective nature also limiting their policy value. More real-time applications of CRC across the operational units engaged in the deployment of CT would merit study. These studies might support the identification and quantification of the impact of operational constraints (e.g., size of contact tracing teams, experienced processes and teams) in the sensitivity of CT. Such efforts to extract more value from CT data might provide additional stimulus to strengthen this critical and neglected public health capacity.

## Conclusion

Capture-recapture models have been used for more than four decades for the estimation of disease surveillance sensitivity. This study provides a relatively simple approach for the estimation of the sensitivity of COVID-19 contact tracing efforts. Completeness of COVID-19 contact tracing in Thailand during the first wave appeared moderate, with around 67% of infectious transmission chains detected. Overdispersion was present suggesting that most of the index cases did not result in infectious transmission chains and the majority of transmission events stemmed from a small proportion of index cases.

## Data Availability

The data that underlie the results reported in this study are available from the corresponding author on reasonable request.

## Appendix: Turing and Chao estimators under Negative Binomial distribution

Under Negative Binomial distribution, the probability function is given by

$$\begin{aligned} p_x =\frac{\Gamma (x+\kappa )}{\Gamma (x+1)\Gamma (\kappa )}\pi ^{\kappa }(1-\pi )^x, x = 0, 1, 2, \ldots \end{aligned}$$

where $\Gamma (.)$ is the Gamma function, and $\pi$ and $\kappa$ are the model parameters. Since $p_0 = \pi ^{\kappa }$, $p_1 = \kappa \pi ^{\kappa }(1-\pi )$ and $E(X) = \kappa (1-\pi )/\pi$. We have $p_1/E(X) = \pi ^{\kappa +1}$ and $p_0 = (\pi ^{\kappa +1})^{\kappa /(\kappa +1)} = (p_1/E(X))^{\kappa /(\kappa +1)}$. In spirit of Turing estimator, we get

$$\begin{aligned} {\hat{N}}_{Turing} = \frac{n}{1-{\hat{P}}_0} = \frac{n}{1-(f_1/S)^{\kappa /(\kappa +1)}}, \end{aligned}$$

where $S = \sum _{x=0}^m xf_x$. In addition, the negative binomial distribution is part of the power series family $p_x = a_xt^xA(t)$ with $a_x = \Gamma (x+\kappa )/(\Gamma (x+1)\Gamma (\kappa ))$ and $A(t) = (1-t)^\kappa$. Considering mixing the negative binomial together with some arbitrary mixing density $\lambda (t)$, we have

$$\begin{aligned} g_x(\lambda |\kappa ) = \int _{0}^{1} \frac{\Gamma (x+\kappa )}{\Gamma (x+1)\Gamma (\kappa )}t^x(1-t)^\kappa \lambda (t) dt = \int _{0}^{1} a_xt^xA(t)\lambda (t)dt. \end{aligned}$$

Since

$$\begin{aligned} \frac{g_1/a_1}{g_0/a_0} \le \frac{g_2/a_2}{g_1/a_1}, \end{aligned}$$

then $a_0$, $a_1$, and $a_2$ are replaced and probabilities are substituted by observed frequencies. For a mixed Negative Binomial model with arbitrary density, the new estimator is accomplished in spirit of Chao estimator as

$$\begin{aligned} {\hat{N}}_{Chao} = n+\frac{(f_1/a_1)^2}{f_2/a_2} = n+\left( \frac{\kappa +1}{\kappa }\right) \frac{f_1^2}{2f_2}, \end{aligned}$$

(see [24]).

## Abbreviations

DDC: Department of disease control
RRT: Rapid response teams
IAR: Intra-action-review
CT: Contact tracing
CRC: Capture-recapture
SS: Surveillance systems
EVD: Ebola virus disease
DRC: Democratic Republic of the Congo
CDCU: Communicable disease control units
JIT: Joint investigation teams
AIC: Akaike information criterion
BIC: Bayesian information criterion
CI: Confidence interval
IQR: Interquartile range
RE: Effective reproductive number
